# Case report: CAR-T therapy demonstrated safety and efficacy in relapsed/refractory diffuse large B-cell lymphoma patients complicated with hepatitis B-related cirrhosis

**DOI:** 10.3389/fonc.2024.1491100

**Published:** 2024-12-05

**Authors:** Danqing Kong, Nana Ping, Qian Zhu, Xiao Zhang, Junhong Li, Rui Zou, Depei Wu, Zhengming Jin, Changju Qu

**Affiliations:** ^1^ National Clinical Research Center for Hematologic Diseases, Jiangsu Institute of Hematology, The First Affiliated Hospital of Soochow University, Suzhou, China; ^2^ Institute of Blood and Marrow Transplantation, Collaborative Innovation Center of Hematology, Suzhou University, Suzhou, China

**Keywords:** diffuse large B-cell lymphoma, cirrhosis, chimeric antigen receptor T-cell therapy, hepatitis B virus, case report

## Abstract

Chimeric antigen receptor T-cell (CAR-T) therapy has demonstrated both efficacy and safety in relapsed/refractory diffuse large B-cell lymphoma (DLBCL) patients infected with hepatitis B virus (HBV). However, its applicability in individuals with liver cirrhosis remains largely unexplored due to the potential for unpredictable complications. Here, we report three cases (P1, P2, and P3) of relapsed/refractory DLBCL with HBV-related cirrhosis treated with CAR-T cell infusion. P1 and P2 received CAR-T cell infusion following a conditioning regimen of fludarabine and cyclophosphamide (FC) for lymphodepletion, while P3 received the SEAM (semustine, etoposide, cytarabine, and melphalan) regimen and autologous stem cell transplantation bridging CAR-T cell infusion. P1 and P2 achieved rapid complete remission (CR), whereas P3 initially exhibited stable disease a month after CAR-T infusion and subsequently achieved CR after local radiation salvage therapy and lenalidomide maintenance. With a median follow-up of 42 months after CAR-T, the progression-free survival rate was 100%. Notably, during follow-up, these patients experienced complications associated with cirrhosis, including endoscopic variceal bleeding, HBV reactivation, or the diagnosis of hepatic malignancy. Our findings suggest that CAR-T therapy is applicable and effective for the treatment of DLBCL patients with HBV-related cirrhosis, albeit necessitating monitoring for potential hepatic complications.

## Introduction

Hepatitis B virus (HBV) infection represents a significant global public health concern, precipitating a cascade of hepatic complications including cirrhosis and hepatocellular carcinoma (HCC), often culminating in fatal outcomes in the absence of timely intervention. Concurrently, diffuse large B-cell lymphoma (DLBCL) stands as one of the predominant B-cell malignancies, characterized by its aggressive nature and challenging treatment landscape. The prevalence of HBV infection in DLBCL patients is higher than that in the general population and often leads to worse clinical outcomes (1, 2). However, there is limited information on treatment recommendations and a lack of appropriate clinical trials for DLBCL patients facing disease progression.

Chimeric antigen receptor T-cell (CAR-T) therapy has been reported as safe and effective in treating relapsed/refractory (R/R) DLBCL and approximately 40% even achieve sustained response (3, 4). Similarly, CAR-T therapy has brought ORR and CR rates of 74.5% and 39.2%, respectively, in our center for DLBCL patients who failed R-CHOP (rituximab, cyclophosphamide, vindesine, adriamycin, and dexamethasone regimen) and other salvage lines of immunochemotherapy (5). Because of the prolonged immunosuppressive state induced by CAR-T therapy, HBV reactivation is one big concern for patients with chronic HBV infection. The risk of HBV reactivation varies according to the patient’s HBV serologic status and treatment modality (6, 7). Recent reports have shown that HBV reactivation occurs in approximately 5.3%–20% of HBsAg-positive patients receiving antiviral prophylaxis after CAR-T therapy, mostly present with asymptomatic increase of HBV DNA copies, and the risk of hepatitis is approximately 5.3% (8–10). Nevertheless, it appears that HBV infection does not adversely affect CAR-T cell viability or efficacy of CAR-T therapy; meanwhile, the incidence and severity of cytokine release syndrome (CRS) and immune effector cell-associated neurotoxicity syndrome (ICANS) do not increase (5, 9, 10).

In 2021, the World Health Organization (WHO) disclosed that among individuals afflicted with chronic HBV infection, up to 40% may progress to cirrhosis if left untreated, with a subset of patients facing the dire consequences of liver malignancy, hepatic failure, or mortality. Liver cirrhosis was also an independent risk factor for HBV reactivation in hematological malignancies (11). Within the subset of patients diagnosed with DLBCL complicated by HBV-related cirrhosis, the utilization of CAR-T therapy had not been reported in the literature. The clinical efficacy and potential adverse events during and after infusion were to be detected. Herein, we presented a single-center case series of R/R DLBCL patients with cirrhosis, who underwent CAR-T cell infusion, delineating both the therapeutic efficacy and associated adverse outcomes.

## Case description

### Patient enrollment and diagnostic criteria

We retrospectively reviewed R/R DLBCL patients who received CAR-T therapy at the First Affiliated Hospital of Soochow University from January 2018 to January 2023. Three DLBCL patients (P1, P2, and P3) complicated with hepatitis B-related cirrhosis were drawn out and analyzed. All patients provided written informed consent. The follow-up visit was conducted until the last visit, June 2024.

The diagnosis of DLBCL was made based on the fifth edition of WHO classification. Chronic HBV infection was defined by the detection of hepatitis B surface antigen (HBsAg) for more than 6 months.

The diagnosis of hepatitis B-related cirrhosis requires a comprehensive approach, involving (1) HBV infection medical history and symptoms such as fatigue or jaundice; (2) laboratory tests including HBV serological tests, HBV-DNA, and liver function; and (3) imaging studies by ultrasonography, computed tomography (CT), or magnetic resonance imaging (MRI) of the liver to detect the signs of cirrhosis and evaluate the complications. The Child–Pugh score system was used to assess the severity of liver disease and predict prognosis. HBV reactivation was defined as at least one of the following: (1) newly detectable HBV-DNA; (2) ≥2 log HBV-DNA increase compared to baseline; and (3) reappearance of HBsAg. The hepatitis flare was defined as alanine aminotransferase (ALT) or aspartate aminotransferase (AST) increased to ≥3 the upper limit of normal. The lower reference limit of HBV-DNA was 60 international units (IU)/milliliter (mL) based on our laboratory standards.

### Patient characteristics and prior immunochemotherapy

All three DLBCL patients had a known history of concomitant HBV infection for years characterized by positive results for HBsAg, but did not pay proper attention. HBV-related cirrhosis was diagnosed through a combination of clinical assessment, laboratory tests, and imaging studies by ultrasonography or CT (see details in **Table 1**). For P1 and P2, cirrhosis was diagnosed at the presentation of DLBCL. P3 was diagnosed with cirrhosis only when the disease relapsed. He was diagnosed with DLBCL and received six cycles of the CHOP regimen, achieving complete remission 5 years ago. At that time, he underwent a short course of antiviral treatment due to chronic HBV infection.

**Table 1:** Characteristics of the three patients at the diagnosis of DLBCL complicated with HBV-related cirrhosis.

Variable	P1	P2	P3
Sex	Male	Male	Female
Age (years)	62	53	46
DLBCL subtype	Non-GCB	Non-GCB	Non-GCB
Ann Arbor stage	IV B	II A	IV B
IPI score	5	1	4
Other high risks	–	Double expression	Double expression Relapsed disease
History of chronic HBV infection	Yes	Yes	Yes
HBV-DNA (range, <ra IU/mL)	3.59×10^7^	<.5	<.5
HBV serology	HBsAg+ HBeAg+ HBcAb+	HBsAg+ HBeAb+ HBcAb+	HBsAg+ HBeAb+ HBcAb+
Hepatic complications besides cirrhosis	Severe ascites; splenomegaly; esophageal varices	Splenomegaly	Esophageal and gastric varices
Child–Pugh grade	Grade B	Grade A	Grade A

DLBCL, diffuse large B-cell lymphoma; HBV, hepatitis B virus; GCB, germinal center B-cell-like; IPI, International Prognostic Index.

**Figure 1:**
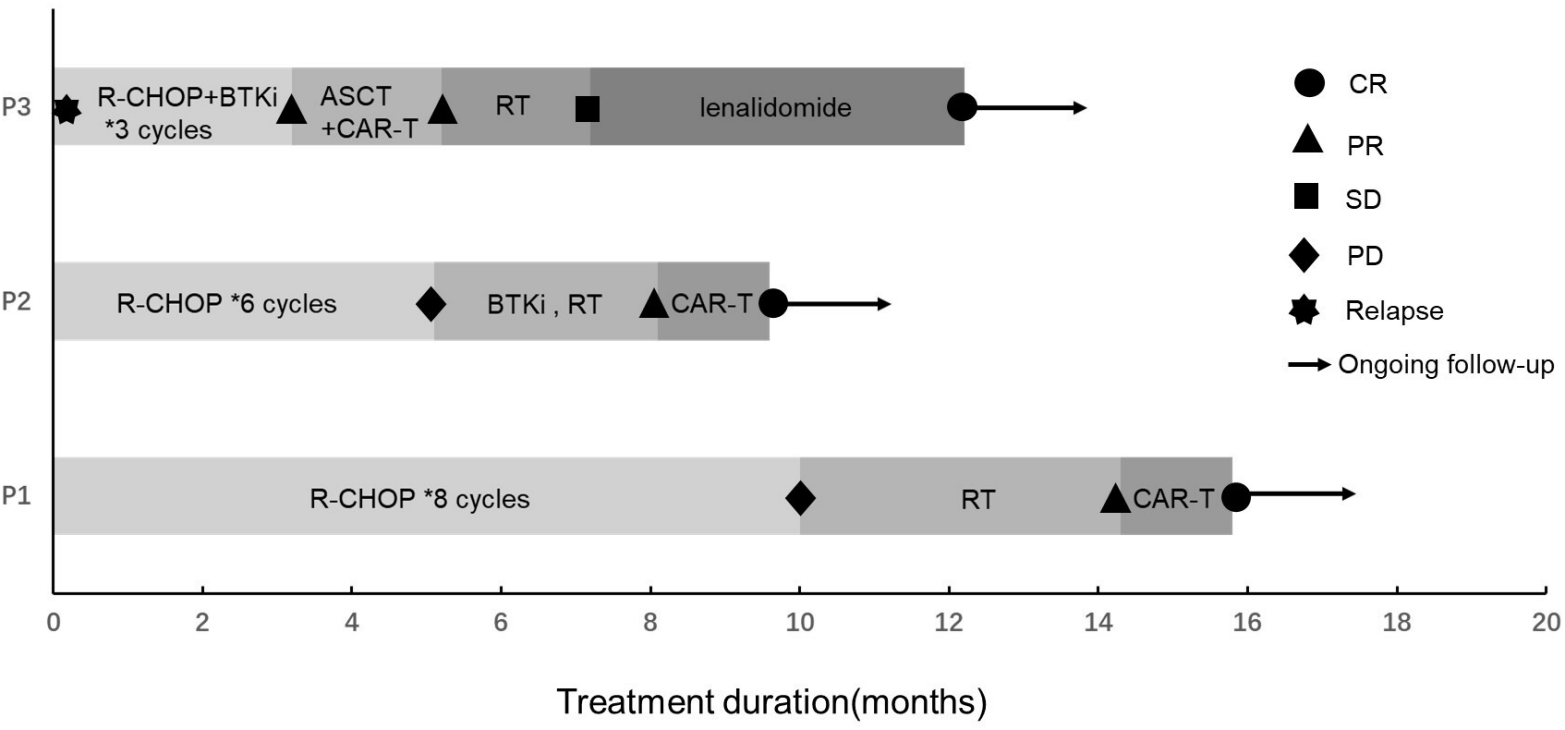
Treatment and response of three DLBCL patients with HBV-related cirrhosis. R-CHOP, rituximab + cyclophosphamide + vindesine + adriamycin + dexamethasone regimen; CAR-T, chimeric antigen receptor T cell; BTKi, Bruton’s tyrosine kinase inhibitor; ASCT, autologous stem cell transplantation; RT, radiotherapy; CR, complete remission; PR, partial remission; SD, stable disease; PD, progressive disease.

They received antiviral treatment with entecavir at a daily dose of 0.5 mg along with supportive measures, and when HBV-DNA was undetectable and liver function improved to a Child–Pugh classification of A, they initiated standard immunochemotherapy without dose reduction, but with split dosing. However, the optimal therapeutic outcomes were not realized, prompting the recommendation for salvage treatment with CAR-T or ASCT bridging CAR-T therapy as salvage treatment. The treatment course and corresponding responses of the patients are summarized in **Figure 1**.

### CAR-T or ASCT bridging CAR-T therapy procedures

CAR-T products were provided by the Unicar-Therapy Bio-Medicine Technology Co. (Shanghai, China), and quality tests were performed before infusion to patients as previously described (12, 13); the detailed procedures are described in **Supplementary Data S1**.

The FC (fludarabine 30 mg/m^2^/d, day −5, −4, and −3; cyclophosphamide 300 mg/m^2^/d, day −5, −4, and −3) conditioning regimen was used for lymphodepletion prior to CAR-T infusion in both P1 and P2. Then, P1 received a scheduled infusion of anti-CD19 and anti-CD22 CAR-T cells at total doses of 1.5×10^7^/kg and 2×10^7^/kg, respectively; P2 received an infusion of tandem CD19/CD22 CAR-T cells at a total dose of 1×10^7^/kg by dose escalation in 3 days (10%, 30%, and 60%). P3 underwent ASCT bridging tandem CD19/CD22 CAR-T therapy. Transplant conditioning used the SEAM (semustine 250 mg/m^2^/d, day −8; etoposide 200 mg/m^2^/d, day −7, −6, −5, and −4; cytarabine 400 mg/m^2^/d, day −7, −6, −5, and −4; melphalan, 140 mg/m^2^/d, day −3) regimen and then autologous stem cells were infused at day 0; the CD34^+^ cell count was 4.12×10^7^/kg. CAR-T cells were infused from day 1 to day 3 at a total dose of 1×10^7^/kg (10%, 30%, and 60%).

The evaluation included complete blood count (CBC), coagulation routine, organ function, expansion of CAR-T cells in peripheral blood, cytokines, and other inflammatory markers such as ferritin.

### Outcome assessment and measurement

Response criteria were defined in accordance with the National Comprehensive Cancer Network (NCCN) guidelines. All adverse events were assessed according to the National Cancer Institute (NCI) Common Terminology Criteria for Adverse Events (CTCAE, Version 4.0). CRS and ICANS were evaluated based on the widely accepted scoring system (14). Progression-free survival (PFS) was calculated from the day of CAR-T infusion to disease progression, relapse, death from any cause, or the last follow-up.

### Clinical efficacy and follow-up of CAR-T therapy or ASCT bridging CAR-T therapy

After CAR-T infusion, positron emission tomography–computed tomography (PET-CT) assessments confirmed complete remission (CR) in P1 and P2. Both individuals received comprehensive clinical oversight from hepatologists and hematologists for concurrent cirrhosis and DLBCL management, resulting in sustained DLBCL remission. P3 exhibited stable disease (SD) 1 month after ASCT bridging CAR-T, subsequently undergoing salvage local radiotherapy, but with minimal effect. Following this, lenalidomide maintenance therapy was initiated 6 months after ASCT bridging CAR-T, remarkably achieving complete disease control, and the patient finally achieved CR at 1 year after ASCT bridging CAR-T and maintained thereafter. Comprehensive radiological evaluations via PET-CT scans of three patients were detailed in **Supplementary Data S2**.

As of June 2024, these three patients had been followed up for a median of 42 months (range, 26.5 to 73 months) after CAR-T infusion; they all achieved sustained PFS. Patients expressed satisfaction with the effectiveness of the CAR-T cell therapy and had an acceptable view of the associated complications detailed below. After undergoing regular checkups and assessments, they were optimistic about the possibility of long-term survival.

### Short-term toxicities

The short-term toxicities associated with CAR-T infusion within a month were mild and reversible in three patients (detailed in **Table 2**). In particular, all showed grade 4 hematologic toxicity, but no CRS ≥grade 3 or ICANS was observed. CD19^+^ B-cell deletion persisted after CAR-T infusion for up to 6 months (range, 4–6 months).

**Table 2:** Short-term toxicities of CAR-T infusion.

Variable	P1	P2	P3
Treatment	CAR-T therapy	CAR-T therapy	ASCT bridging CAR-T therapy
CRS	1	0	2
ICANS	0	0	0
Fever	Yes	None	Yes
Hypotension	None	None	None
Hypoxemia	None	None	Low-flow oxygen
ALT/AST ≥ 3 ULT	None	None	Yes
Total bilirubin ≥ 2 ULT	None	None	None
Albumin<lb g/L	None	None	None
Prolonged coagulation	None	None	None
Hematologic toxicity	Grade 4	Grade 4	Grade 4
IL-6 max/baseline	20.6	2.2	150.7
Other short-term toxicities	None	None	Gastrointestinal mucositis; upper gastrointestinal hemorrhage

CAR-T, chimeric antigen receptor T cell; ASCT, autologous stem cell transplantation; CRS, cytokine release syndrome; ICANS, immune effector cell-associated neurotoxicity syndrome; ALT, alanine transaminase; AST, aspartate transaminase; ULN, upper limit of normal.

### Long-term hepatic complications

All patients received indefinite antiviral treatment with entecavir. Laboratory tests indicated a persistent HBsAg-positive, Child–Pugh classification of A ever since induction therapy. They also maintained undetectable HBV-DNA throughout the whole episode except P1.

**Figure 2:**
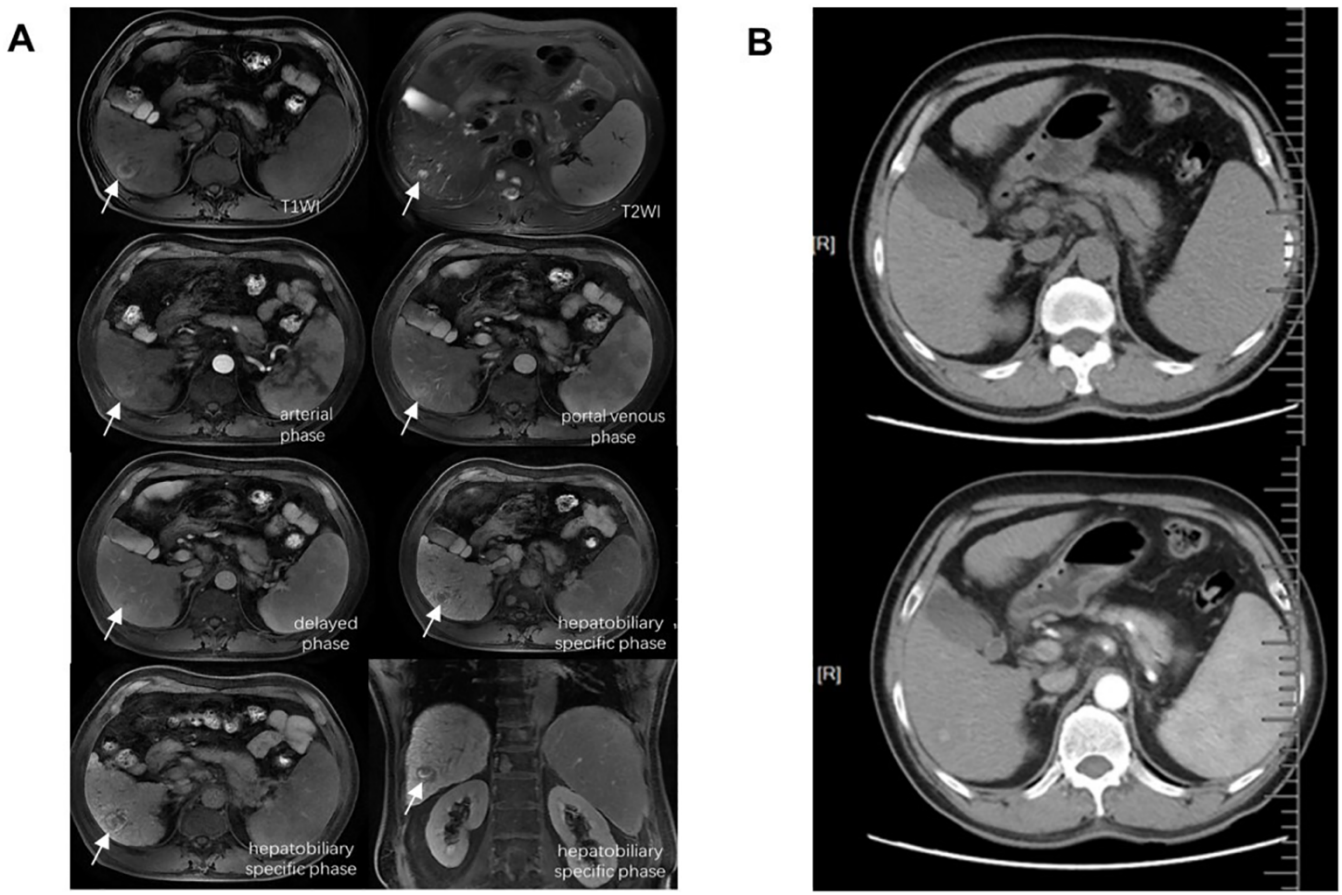
Major radiographic assessments of P1 for the diagnosis of liver malignant tumor. **(A)** Enhanced MRI by Gd-EOB-DTPA. **(B)** Enhanced CT scan. Both revealed newly detected nodules with a maximum diameter exceeding 2 cm. MRI, magnetic resonance imaging; CT, computed tomography.

P1 was diagnosed as hepatic malignancy based on typical radiographic signs that both enhanced CT and MRI scans revealed newly detected nodules with a maximum diameter exceeding 2 cm 14 months after CAR-T therapy (**Figure 2**). Laboratory assessments indicated HBV reactivation, with an HBV-DNA level of 94.4 IU/mL and elevated serum bilirubin levels of 36.4 μmol/L, suggestive of liver function impairment. Serum alpha-fetoprotein (AFP), AST, ALT, platelet counts, and coagulation tests remained within normal range while Child–Pugh grade maintained an A classification. The patient refused surgical intervention or chemotherapy/radiotherapy options due to concerns about adverse reactions, and he opted for continuous antiviral therapy with entecavir and short-term adefovir, coupled with several sessions of transcatheter arterial chemoembolization (TACE), ablation therapy, and three cycles of anti-PD-1 inhibitor camrelizumab administration. Throughout his last visit, the patient remained alive with liver neoplasm under good control and undetectable HBV-DNA (the trend of HBV-DNA copies is shown in **Supplementary Data S3**).

P3 underwent indefinite lenalidomide maintenance therapy to manage DLBCL, maintaining platelet levels between 50 and 100×10^9^/L. However, the patient experienced two episodes of esophagogastric variceal bleeding. She underwent endoscopic tissue glue injection and variceal ligation surgeries successively, additionally with supporting treatments such as fasting, proton pump inhibitors (PPIs), pancreatic secretion inhibition, and nutritional support. Her condition remained stable and under control until the last follow-up assessment.

## Discussion

DLBCL patients with chronic HBV infection revealed distinct clinical features of a younger age, more advanced disease stage, and even poorer outcomes (1, 2). The role of HBV in the mechanism of DLBCL had not been fully elucidated; one hypothesis was that, like hepatitis C, a protracted antigenic stimulation of B cells produced virus-specific antibodies; another hypothesis was based on genome-wide analysis, which revealed that enhanced gene mutation load possibly mediated by apolipoprotein B mRNA-editing enzyme catalytic polypeptide (APOBEC) enzyme activity and the activity of the B-cell-specific activation induced cytidine deaminase (AID) (1, 15).

Currently, there were no specific guidelines or sufficient clinical trials available for treating R/R DLBCL with HBV infection. HBV X protein (HBX) overexpressed in DLBCL with HBV infection and HBX expression potentiated resistance to S-phase arrest-inducing chemotherapeutics like methotrexate (MTX) and cytarabine (Ara-C) *in vitro* (16, 17), suggesting that neither MTX nor Ara-C was suitable as second-line treatments. CAR-T therapy had been attempted in this disease entity under prophylactic antiviral therapy (8–10). In a study of 15 R/R DLBCL patients with HBV infection, overall response (OR) and CR rate of CAR-T therapy was 66.7% and 60%, respectively, accompanying controllable CRS. HBV reactivation was seen in three patients (9).

However, DLBCL patients with HBV-related cirrhosis have received scant attention in medical literature. A retrospective case series of eight patients assessed the administration of rituximab in DLBCL patients with HBV-related cirrhosis, all of whom were undergoing standardized antiviral therapy. Among them, one patient experienced HBV reactivation, yet neither hepatitis flares nor abnormal liver function was observed. The median overall survival (OS) was 39 months (range, 7–82 months), indicating that rituximab was a safe option for these patients (18). In our study, the CR rate was 66.7%, and P3 did not achieve the expected efficacy despite receiving more intensive treatment with ASCT bridging CAR-T. HBV reactivation occurred in P1 despite whole-course combination of entecavir prophylaxis. The crucial risk factors for HBV reactivation can be broadly classified into three categories: host-related factors, virus-related factors, and immunosuppressive therapy. Immunosuppressive therapy is the major risk and quite common applied in DLBCL patients. In oncological practice, it is recommended to initiate antiviral prophylaxis with nucleoside analogs (NAs) before immunosuppressive therapies. Compared to the first approved NA lamivudine, the next-generation NA entecavir is less likely to cause drug resistance and is more effective in viral suppression and in preventing HBV reactivation among HBsAg-positive lymphoma patients undergoing chemotherapy (19). It is reported that tenofovir may be more effective than entecavir in HBeAg-positive patients (20). Entecavir and tenofovir are now recommended standard agents for the prevention and treatment of HBV reactivation in patients under immunosuppressive therapy. In the case of P1, entecavir was quite effective, but considering the transient detectable HBV-DNA presenting without drug withdrawal, entecavir was not a guarantee for avoiding HBV reactivation.

CRS is a major concern since chronic HBV infection may induce higher IL-6 production (21). Neither our cases nor other reported patients with HBV infection observed an increase in the frequency or severity of CRS or ICANS. However, owing to the constraint of a small sample size, the relationship between HBV infection and CRS warrants further investigation.

The clinical presentation of HBV reactivation or HBV-related mortality varied, ranging from unnoticed elevated HBV-DNA levels and/or ALT to life-threatening fulminant hepatitis, hepatic encephalopathy, and liver failure (9, 10, 22). However, the occurrence of newly developed hepatic malignancy was rare. In chronic viral hepatitis-related cirrhosis patients, the estimated cumulative incidence of HCC over a 10-year period was only approximately 4.0% (23). HBV plays a direct role in HCC transformation by triggering specific oncogenic pathways as well as stimulating host immune response and driving liver chronic necro-inflammation (24). Furthermore, complex immune imbalance may promote HCC transformation (25). In P1, despite the potential for CAR-T therapy to induce immune dysregulation characterized by cytokine elevation and B lymphocyte abnormalities, the likelihood of it significantly contributing to HCC transformation appears low. This conclusion is supported by the relatively modest and transient elevation in cytokine levels post-therapy, as well as the restoration of B lymphocyte counts within 6 months. Overall, it is preferable to believe that CAR-T therapy is not primarily responsible for hepatic malignancy development. The occurrence of cirrhosis cancerization following CAR-T therapy in this case may be coincidental; however, caution should still be exercised regarding this issue.

Recent studies have focused on the evaluation of ASCT bridging CAR-T therapy vs. CAR-T therapy for R/R DLBCL. One single-arm prospective clinical study (26) explored the efficacy and safety of ASCT bridging CD19/CD22 CAR-T therapy in 42 R/R aggressive B-cell non-Hodgkin lymphoma (B-NHL) patients, at a median follow-up of 24.3 months, the OR rate was 90.5%, the 2-year PFS rate was 83.3%, and the median PFS and OS were not reached. All cases of CRS and ICANS were reversible. Another retrospective cohort study (27) compared ASCT-CART to ASCT in 67 R/R DLBCL patients. The ASCT-CART group showed a higher CR rate (71% vs. 33%; *p* = 0.003), a superior 3-year PFS (80% vs. 44%; *p* = 0.036), and a lower 3-year relapse/progression rate (15% vs. 56%; *p* = 0.015) than the ASCT group. Although P3 in our study failed this combined therapy, the prospect of future trials to explore the efficacy and safety of ASCT bridging CAR-T therapy in a larger patient cohort is still promising. P3 achieved CR under lenalidomide maintenance 6 months after ASCT bridging CAR-T. Lenalidomide, an oral immunomodulatory drug, exerts its effects primarily through direct anti-tumor activity, immune modulation, and regulation of the tumor microenvironment (28). We conducted a study (29) evaluating lenalidomide maintenance after CAR-T therapy in R/R DLBCL patients, and we found that OS was significantly prolonged in the lenalidomide maintenance group; in addition, the *in vitro* test showed that the delayed exhaustion of CAR-T cells may contribute to the OS benefit. The remission observed in P3 cannot be attributed to delayed exhaustion of CAR-T cells, as these cells were undetectable prior to maintenance therapy. Nonetheless, the early introduction of lenalidomide maintenance therapy following CAR-T treatment may enhance efficacy and promote sustained peripheral blood levels of CAR-T cells. Looking back retrospectively, gene next-generation sequencing (NGS) could not be performed and many novel targeting drugs were not available; otherwise, genetic guided targeting treatment might have been an alternative choice for these patients.

There are some limitations in our report. The number of cases included is relatively small. Since the incidence rate of R/R DLBCL combined with cirrhosis is low, and the majority of the patients are CAR-T therapy ineligible due to patients’ physical limitations and high cost, there is difficulty in finding more candidates. Additionally, follow-up is still required to assess long-term efficacy and complications. In general, CAR-T therapy demonstrates effectiveness with tolerable treatment-related toxicities in treating our three refractory DLBCL patients with HBV-related cirrhosis. However, concerns persist regarding HBV reactivation and other long-term complications, despite the use of antiviral prophylaxis. Close monitoring of HBV-DNA levels, liver imaging, and liver function after CAR-T therapy is imperative. Further studies and data collection are warranted to ascertain whether CAR-T therapy is a suitable strategy for this unique patient population.

## Data Availability

The original contributions presented in the study are included in the article/**Supplementary Material**. Further inquiries can be directed to the corresponding authors.
