# Supplementary material for: Case report: CAR-T therapy demonstrated safety and efficacy in relapsed/refractory diffuse large B-cell lymphoma patients complicated with hepatitis B-related cirrhosis
Source: Front Oncol. 2024 Dec 5;14:1491100. doi: 10.3389/fonc.2024.1491100 (PMC11655506; doi:10.3389/fonc.2024.1491100)
Supplement: Supplementary file 1 [file DataSheet1.docx]

**S1 CAR-T Cell Preparation steps**

The peripheral blood lymphocytes were acquired through density gradient separation from patients’ peripheral blood collected by apheresis. T lymphocytes were further collected and separated from peripheral blood lymphocytes by degradable anti-CD3 magnetic microbeads (Miltenyi Biotec,

Bergisch Gladbach, Germany) and activated with 5 μg/mL CD3 and CD28 (Miltenyi Biotec) for 18–24 hours. Thereafter, the T cells were transduced with recombinant lentiviral vectors, containing the anti-CD19/22 single-chain variable fragment (scFv), the cytoplasmic portion of the 4-1BB costimulatory moiety, and the CD3z T-cell activation domain, by adding lentivirus directly to the medium. After 48 hours of lentiviral transduction, the cells were washed and expanded with the complete medium (X-vivo 15 medium; Lonza, GA) containing 5 ng/mL interleukin (IL)-7 (Peprotech, NJ,USA) and 5 ng/mL IL-15 (Peprotech, NJ) cytokines under a 37°C, 5% CO2 humidified condition and maintained at a concentration of 0.5×10^6^ cells/mL for 12–20 days until their numbers met the preset value. Finally, the second-generation CAR-T cells targeting CD19, CD22 or tandem CD19/CD22 were harvested, washed with 0.9% saline, and concentrated in cryopreservation followed by quality tests including cell viability, transduction efficiency, purity, killing ability in vitro, cytokine release capacity, quantity of endotoxin, mycoplasma, bacteria, fungus, and so on before infusion to patients. Acceptance criteria for CAR-T cell infusion were as follows: (1) Trypan blue viability > 90%, (2) CAR transduction efficiency > 10%, (3) purity of CD3+ cell > 95%, CD19+ cell: negative, (4) killing ability in vitro > 10%, (5) cytokine release capacity > 10%, (6) Gram stain: negative, endotoxin <3 EU/mL, (7) mycoplasma (polymerase chain reaction): negative, and (8) fungus: negative. In this study, the amplified folds of total cell number ranged from 50 to 100, and the proportion of CAR expressed cells in the final cell products ranged from 30% to 70%. All these CAR-T products were provided by the UnicarTherapy Bio-medicine Technology Co.

**S2 Major radiographic assessments of the patients**


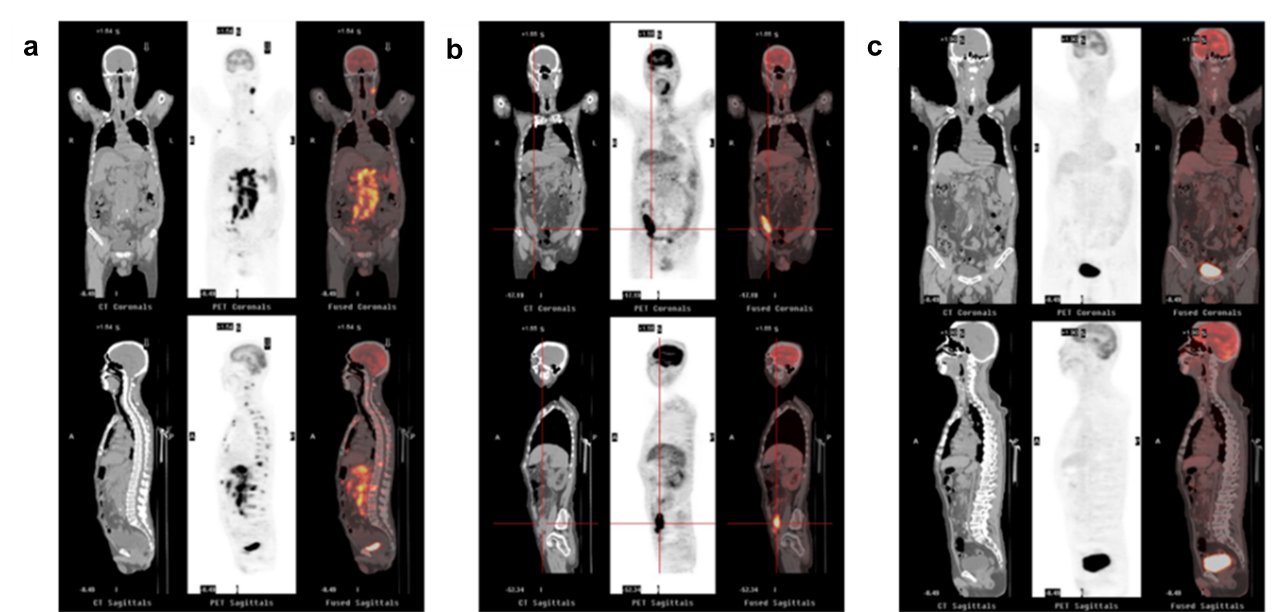


Figure 1 PET-CT imaging dynamic changes of patient 1

(A) PET-CT at diagnosis showed high SUV of duodenum, multiple enlarged lymph nodes and bones. (B) PET-CT after R-CHOP indicated less involved lymph nodes and bones, but newly discovered invasion of terminal ileum. (C) PET-CT after CAR-T therapy indicated complete metabolic response of DLBCL.


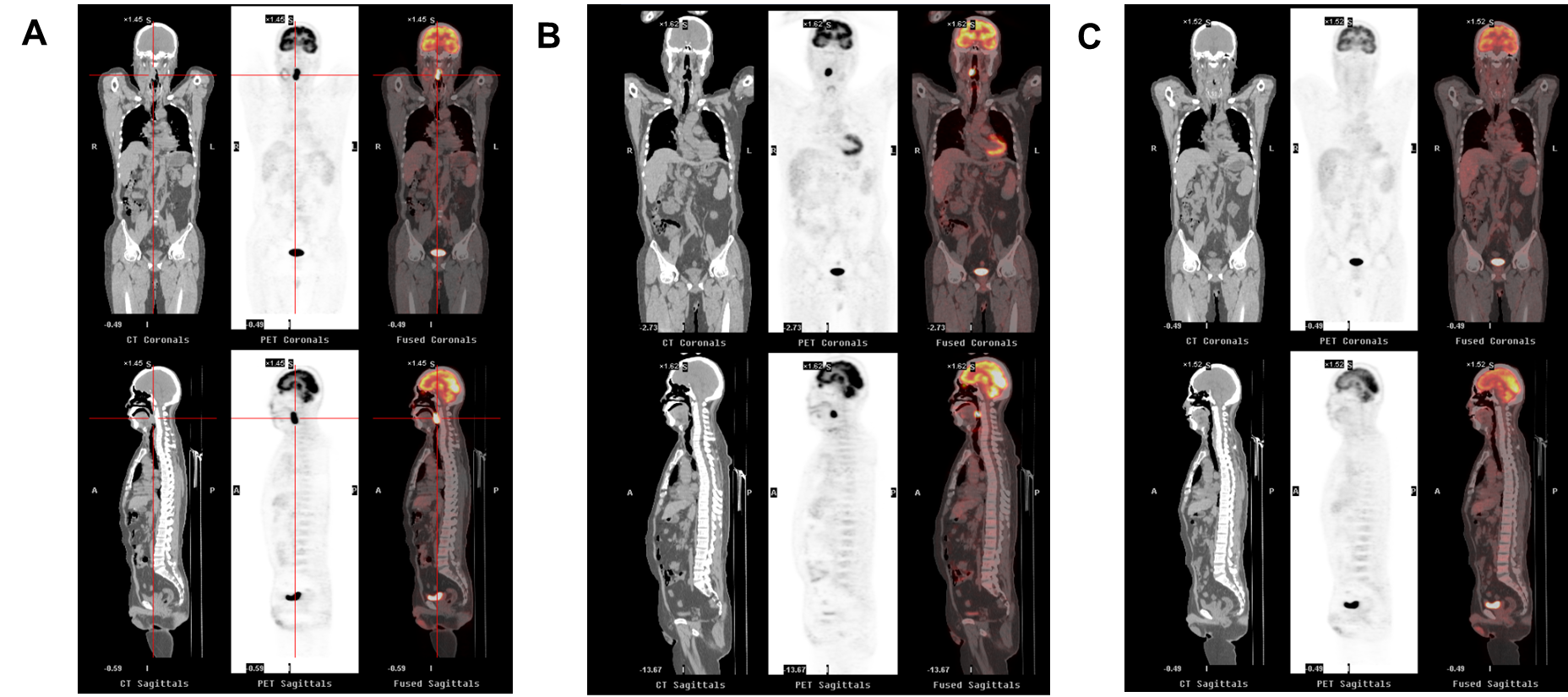


Figure 2 PET-CT imaging dynamic changes of patient 2

(A) PET-CT at diagnosis showed enlarged lymph nodes with increased glucose metabolism in the right neck and right side of the oropharynx. (B) PET-CT after R-CHOP revealed thickening in the right oropharynx with higher SUV. (C) PET-CT after CAR-T therapy showed complete metabolic response of DLBCL.


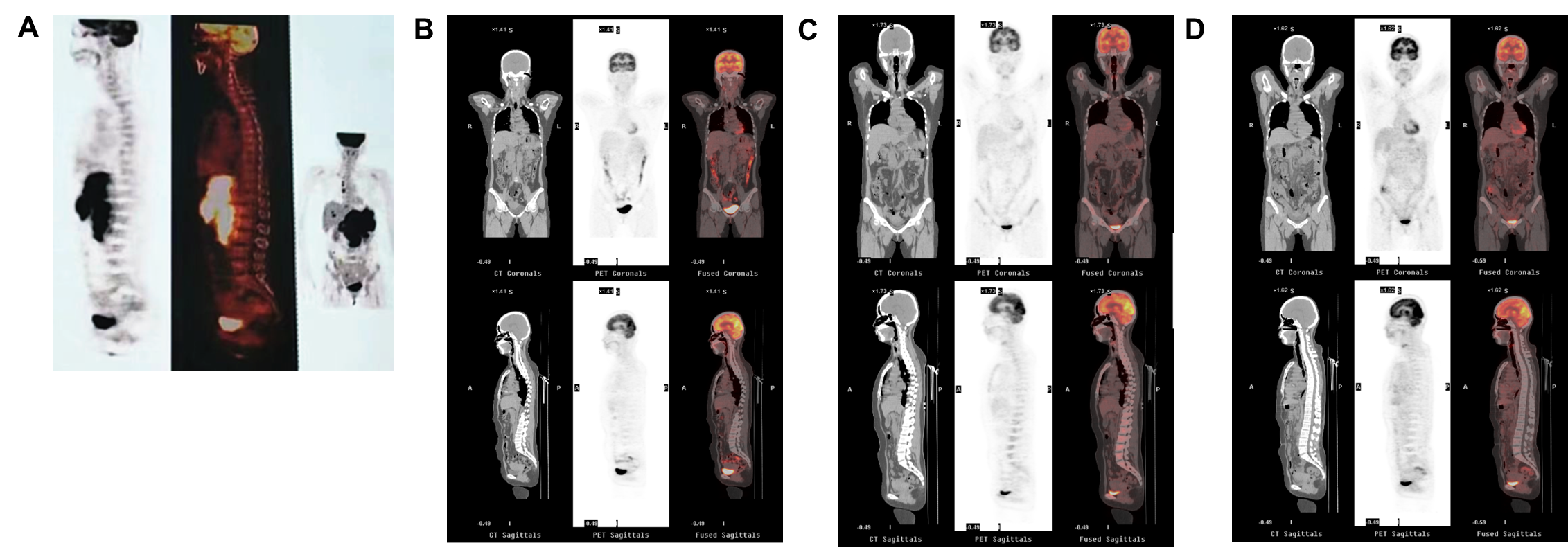


Figure 3 PET-CT imaging dynamic changes of patient 3

(A) PET-CT at relapse showed a large mass in abdomen with infiltration into surrounding organs, multiple enlarged lymph nodes on both sides of the diaphragm. (B) PET-CT after R-CHOP showed the abdominal mass had decreased in size by over 50% compared to previous measurements and reached partial remission response. (C) PET-CT after CAR-T therapy indicated stable disease of the abdominal mass. (D) PET-CT after lenalidomide maintenance showed complete metabolic response of DLBCL.

PET-CT, positron emission tomography-computed tomography; SUV, standard uptake value; R-CHOP, rituximab+ cyclophosphamide+ vindesine+ adriamycin+ dexamethasone regimen; CAR-T, chimeric antigen receptor T cell; DLBCL, diffuse large B-cell lymphoma

**S3 Treatment, antiviral therapy, and trend of HBV-DNA copies in patient 1.**
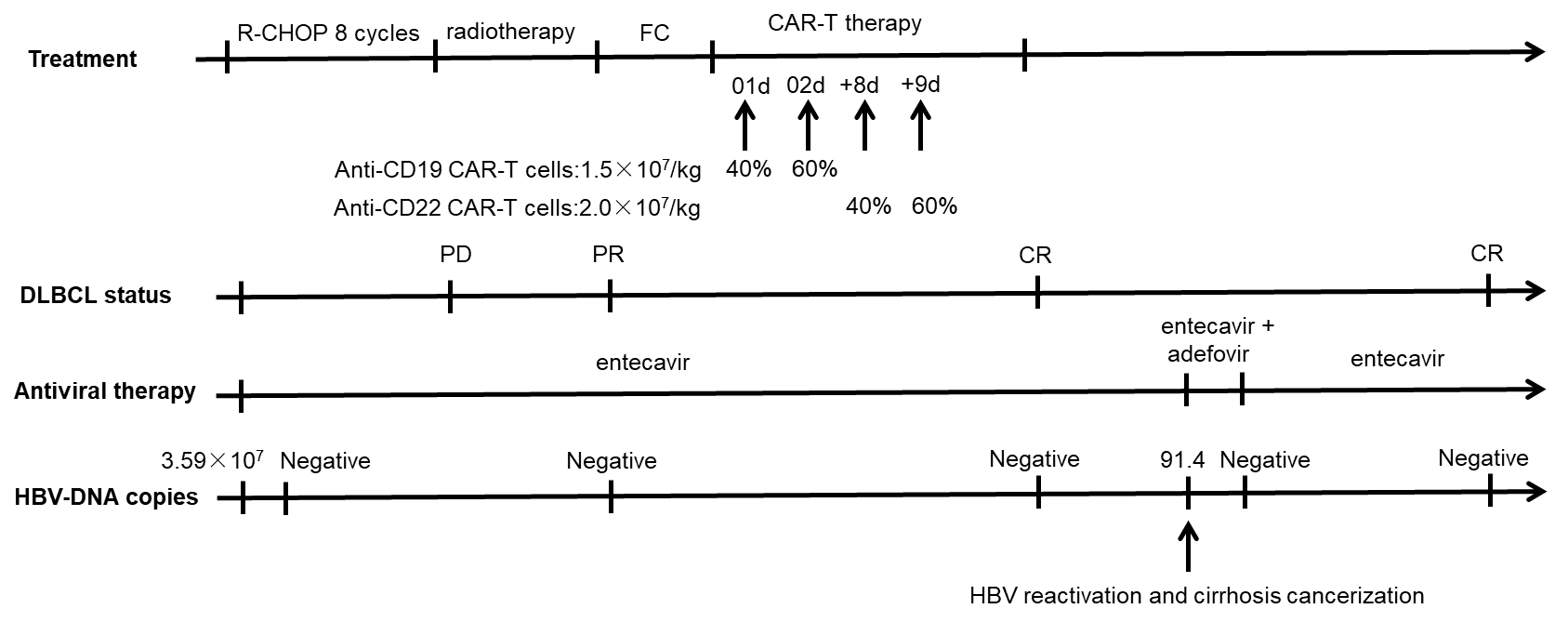


Figure 4 Treatment, antiviral therapy, and trend of HBV-DNA copies in patient 1.

Note: R-CHOP, rituximab+cyclophosphamide+vindesine+adriamycin

+dexamethasone regimen; FC, fludarabine+cyclophosphamide regimen; CAR-T, chimeric antigen receptor T cell; DLBCL, diffuse large B-cell lymphoma; PD, progressive disease; PR, partial remission; CR, complete remission.
